# Temporal Trends in the Use of Therapeutic Hypothermia for Out-of-Hospital Cardiac Arrest

**DOI:** 10.1001/jamanetworkopen.2018.4511

**Published:** 2018-11-09

**Authors:** Steven M. Bradley, Wenhui Liu, Bryan McNally, Kimberly Vellano, Timothy D. Henry, Michael R. Mooney, M. Nicholas Burke, Emmanouil S. Brilakis, Gary K. Grunwald, Mehul Adhaduk, Michael Donnino, Saket Girotra

**Affiliations:** 1 Minneapolis Heart Institute, Minneapolis, Minnesota; 2 Minneapolis Heart Institute Foundation, Minneapolis, Minnesota; 3 Veterans Affairs Eastern Colorado Health Care System, Denver; 4 University of Colorado School of Public Health, Aurora; 5 Emory University School of Medicine, Rollins School of Public Health, Atlanta, Georgia; 6 Cedars-Sinai Medical Center, Los Angeles, California; 7 University of Iowa Carver College of Medicine, Iowa City; 8 Beth Israel Deaconess Medical Center, Boston, Massachusetts

## Abstract

**Importance:**

Despite evidence that therapeutic hypothermia improves patient outcomes for out-of-hospital cardiac arrest, use of this therapy remains low.

**Objective:**

To determine whether the use of therapeutic hypothermia and patient outcomes have changed after publication of the Targeted Temperature Management trial on December 5, 2013, which supported more lenient temperature management for out-of-hospital cardiac arrest.

**Design, Setting, and Participants:**

A retrospective cohort was conducted between January 1, 2013, and December 31, 2016, of 45 935 US patients in the Cardiac Arrest Registry to Enhance Survival who experienced out-of-hospital cardiac arrest and survived to hospital admission.

**Exposures:**

Calendar time by quarter year.

**Main Outcomes and Measures:**

Use of therapeutic hypothermia and patient survival to hospital discharge.

**Results:**

Among 45 935 patients (17 515 women and 28 420 men; mean [SD] age, 59.3 [18.3] years) who experienced out-of-hospital cardiac arrest and survived to admission at 649 US hospitals, overall use of therapeutic hypothermia during the study period was 46.4%. In unadjusted analyses, the use of therapeutic hypothermia dropped from 52.5% in the last quarter of 2013 to 46.0% in the first quarter of 2014 after the December 2013 publication of the Targeted Temperature Management trial. Use of therapeutic hypothermia remained at or below 46.5% through 2016. In segmented hierarchical logistic regression analysis, the risk-adjusted odds of use of therapeutic hypothermia was 18% lower in the first quarter of 2014 compared with the last quarter of 2013 (odds ratio, 0.82; 95% CI, 0.71-0.94; *P* = .006). Similar point-estimate changes over time were observed in analyses stratified by presenting rhythm of ventricular tachycardia or ventricular fibrillation (odds ratio, 0.89; 95% CI, 0.71-1.13, *P* = .35) and pulseless electrical activity or asystole (odds ratio, 0.75; 95% CI, 0.63-0.89; *P* = .001). Overall risk-adjusted patient survival was 36.9% in 2013, 37.5% in 2014, 34.8% in 2015, and 34.3% in 2016 (*P* < .001 for trend). In mediation analysis, temporal trends in use of hypothermia did not consistently explain trends in patient survival.

**Conclusions and Relevance:**

In a US registry of patients who experienced out-of-hospital cardiac arrest, the use of guideline-recommended therapeutic hypothermia decreased after publication of the Targeted Temperature Management trial, which supported more lenient temperature thresholds. Concurrent with this change, survival among patients admitted to the hospital decreased, but was not mediated by use of hypothermia.

## Key Points

**Question:**

Did the use of therapeutic hypothermia for out-of-hospital cardiac arrest change after publication of a trial that supported more lenient temperature goals?

**Findings:**

In a cohort study of 45 935 US patients who experienced out-of-hospital cardiac arrest and survived to hospital admission, the risk-adjusted odds of the use of therapeutic hypothermia decreased 18% in the first quarter of 2014 after publication of the Targeted Temperature Management trial, compared with the last quarter of 2013. Risk-adjusted patient survival also decreased during the study period, but was not mediated by trends in hypothermia use.

**Meaning:**

In a US registry of patients who experienced out-of-hospital cardiac arrest, the use of guideline-recommended active cooling decreased after publication of the Targeted Temperature Management trial.

## Teaser

This cohort study examines whether the use of therapeutic hypothermia and patient survival have changed after publication of the Targeted Temperature Management trial, which supported more lenient temperature management for out-of-hospital cardiac arrest.

## Introduction

Therapeutic hypothermia is a guideline-recommended therapy for patients who remain comatose after resuscitation of out-of-hospital cardiac arrest. These recommendations are based largely on 2 randomized trials that demonstrated survival benefit with therapeutic hypothermia to a target temperature of 32°C to 34°C.^1,2^ However, debate remains as to whether these findings reflect the benefit of hypothermia or the harm of fever in the control group of prior studies.^2,3^

A third trial was published in December 2013 that investigated the effect of 2 different temperature goals, both intended to avoid fever, on patient outcomes after out-of-hospital cardiac arrest.^4^ Active measures (eg, surface cooling, ice packs, intravascular catheters) were used in both treatment groups to achieve temperature goals. This Targeted Temperature Management (TTM) trial observed similar outcomes among patients treated with therapeutic hypothermia to a target temperature of 36°C as compared with a target temperature of 33°C. These findings suggest that a more lenient temperature threshold for therapeutic hypothermia is reasonable in the management of out-of-hospital cardiac arrest.

It is unknown how the findings of the TTM trial have been applied in routine clinical practice. Given the trial’s emphasis on the use of hypothermia for fever avoidance, use of therapeutic hypothermia in clinical practice may be declining in favor of other untested approaches to fever avoidance in the management of out-of-hospital arrest (ie, administration of acetaminophen). Alternatively, the more lenient temperature goals supported by the TTM trial may have encouraged greater use of therapeutic hypothermia in clinical practice. Finally, the implications on patient outcomes in real-world practice have not been explored, to our knowledge.

To address this gap in knowledge, we examined temporal trends in the use of therapeutic hypothermia among patients who experienced out-of-hospital cardiac arrest and survived to hospital admission in the Cardiac Arrest Registry to Enhance Survival (CARES). Given that the epidemiologic characteristics of presenting cardiac arrest rhythm have changed over time, with a decline in cardiac arrests with ventricular tachycardia (VT) or ventricular fibrillation (VF), and clinical trial evidence supporting therapeutic hypothermia is strongest for cardiac arrest with VT or VF, we evaluated overall trends in use of therapeutic hypothermia and also conducted stratified analyses on presenting arrest rhythm. Finally, we explored the association between patient-level and hospital-level trends in the use of therapeutic hypothermia and risk-adjusted patient survival. Understanding trends in the use of therapeutic hypothermia and associated patient outcomes may inform future strategies to achieve optimal use of this guideline-recommended therapy.

## Methods

### Data Source and Study Population

The CARES registry is a large, prospective clinical registry established by the Centers for Disease Control and Prevention and Emory University for surveillance and quality improvement in the care of patients who experienced out-of-hospital cardiac arrest. The design of the registry has been previously described.^5,6^ Briefly, patients with confirmed out-of-hospital cardiac arrest (defined as apneic and unresponsive) of nontraumatic cause and for whom resuscitation is attempted are identified. Data on these patients are collected from 911 dispatch centers, emergency medical services agencies, and receiving hospitals using standardized international Utstein definitions^5,6^ for clinical variables and outcomes with review of records by a CARES analyst to ensure completeness and internal consistency. This study followed the Strengthening the Reporting of Observational Studies in Epidemiology (STROBE) guidelines for observational studies.^7^ The Colorado Multiple Institutional Review Board approved this study and a waiver of informed consent was granted.

**Figure 1.**
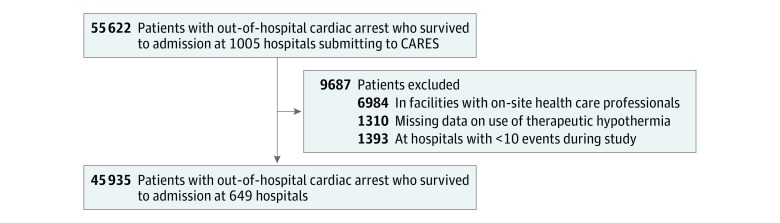
Identification of Patient Cohort CARES indicates Cardiac Arrest Registry to Enhance Survival.

We identified 55 622 patients whose cases were submitted to CARES between January 1, 2013, and December 31, 2016, who survived to admission at 1005 hospitals and thus were eligible for therapeutic hypothermia on admission (Figure 1). We excluded 6984 patients (12.6%) with cardiac arrests that occurred in facilities with on-site health care professionals (ie, nursing home, clinic, or hospitals) and 1310 patients (2.4%) with missing data on the use of therapeutic hypothermia. Furthermore, we excluded 1393 patients (2.5%) from 356 hospitals with fewer than 10 events in CARES during the study period to allow for stable estimates in our analyses that accounted for clustering of patients in hospitals. The final cohort comprised 45 935 patients.

### Independent Variable and Study Outcome

The independent variable was calendar time by quarter year, which was evaluated as a categorical variable. The primary outcome of interest was the use of therapeutic hypothermia on admission to the hospital. *Therapeutic hypothermia* is defined in the CARES registry as the provision of measures to reduce the patient’s body temperature by either noninvasive means (eg, administration of cold intravenous saline; external cold pack application to armpits or groin; or use of a cooling blanket, torso vest, or leg wrap devices) or invasive means (eg, use of a cooling catheter inserted via the internal jugular, subclavian, or femoral vein). Because trends in presenting cardiac arrest rhythm are known to be changing and the trial evidence for therapeutic hypothermia is largest for VT or VF, we analyzed temporal trends in the use of therapeutic hypothermia for the overall cohort and separately for VT or VF and pulseless electrical activity (PEA) or asystole.

In secondary analyses, we sought to evaluate the extent to which temporal trends in survival to hospital discharge were mediated by trends in use of hypothermia. Because use of hypothermia and survival of cardiac arrest were both anticipated to change over time, we used causal mediation analysis to disentangle trends in patient survival that were directly attributable to temporal trends vs those mediated by changing use of hypothermia over time (eAppendix in the Supplement).^8^ We performed these analyses at both the patient level and hospital level. Details on the methods of this analysis are provided in the eAppendix in the Supplement. In these analyses, we restricted the cohort to the 300 hospitals that participated in the CARES registry for all 4 years of the study period to ensure that findings were robust to changes in participating hospital characteristics and we restricted the cohort to hospitals with more than 5 events per year to avoid inflation of variance due to small numbers.

### Statistical Analysis

We tested the trends of patient characteristics (age, sex, and race/ethnicity), cardiac arrest characteristics (initial rhythm, location of cardiac arrest, and witnessed), bystander response (bystander cardiopulmonary resuscitation and bystander use of automated external defibrillator), and characteristics of emergency medical services (response time, placement of advanced airway, and hypothermia in the field) over calendar years. A univariate model was fit for each variable, with linear function of calendar year as the predictor; *F* tests were used for polytomous variables, and *t* tests were used for continuous and binary variables. All *P* values were from 2-sided tests and results were deemed statistically significant at *P* < .05. With the exception of race/ethnicity (25% missing) and use of advanced airway (16% missing), covariates were complete. Missingness was not at random as some hospitals and emergency medical services agencies did not collect this information. Accordingly, we chose to use indicator variables when accounting for missing covariates in risk adjustment.

We describe the unadjusted proportion of patients treated with therapeutic hypothermia by quarter-year for the overall cohort and by initial presenting rhythm (VT or VF, or PEA or asystole). To assess whether use of therapeutic hypothermia has changed over time, we first fit a generalized linear mixed model with quarter-year as a categorical variable to compare the use of therapeutic hypothermia at each time point of analysis. We then conducted interrupted time series analysis using generalized linear mixed models to assess risk-adjusted trends in the use of therapeutic hypothermia before and after publication of the TTM trial in December 2013. These mixed-effects models included covariates for patient characteristics, bystander response, and emergency medical services treatment as described in Table 1 and random intercepts for hospital to account for clustering of patients within hospitals and to account for different hospitals joining CARES at different time points. Covariates were selected in accordance with prior literature and no model comparisons were done for this study.^9^ We performed these analyses on the overall cohort and stratified by initial cardiac arrest rhythm (VT or VF, or PEA or asystole) and fit a model for the interaction of presenting rhythm and change of hypothermia intercept and slope. In sensitivity analyses, we restricted our cohort to the 300 hospitals with continuous participation in the CARES registry to avoid trends reflecting changes in the practice of participating hospitals rather than change in clinical practice. These analyses were done with SAS statistical software, version 9.4 (SAS Institute Inc).

**Table 1.** Patient and Arrest Characteristics Over Time

Characteristic	Patients, No. (%)					*P* Value for Trend
	Overall (N = 45 935)	2013 (n = 8119)	2014 (n = 10 512)	2015 (n = 12 591)	2016 (n = 14 713)	
Age, mean (SD), y	59.3 (18.3)	59.8 (18.3)	59.7 (18.2)	59.0 (18.4)	58.9 (18.3)	<.001
Male	28 420 (61.9)	4956 (61.0)	6504 (61.9)	7794 (61.9)	9166 (62.3)	.08
Race/ethnicity						
White	21 831 (47.5)	3602 (44.4)	5031 (47.9)	6172 (49.0)	7026 (47.8)	<.001
Black	8196 (17.8)	1461 (18.0)	1841 (17.5)	2252 (17.9)	2642 (18.0)	
Hispanic/Latino	2902 (6.3)	618 (7.6)	685 (6.5)	778 (6.2)	821 (5.6)	
Unknown	11 501 (25.0)	2162 (26.6)	2608 (24.8)	2964 (23.5)	3767 (25.6)	
Other	1503 (3.3)	274 (3.4)	347 (3.3)	425 (3.4)	457 (3.1)	
Witnessed arrest	31 052 (67.6)	5490 (67.6)	7143 (68.0)	8468 (67.3)	9951 (67.6)	.75
Location of arrest						
Residence	34 042 (74.1)	6074 (74.8)	7826 (74.4)	9330 (74.1)	10 812 (73.5)	.002
Street or highway	3751 (8.2)	588 (7.2)	892 (8.5)	992 (7.9)	1279 (8.7)	
Public or commercial building	5618 (12.2)	963 (11.9)	1242 (11.8)	1586 (12.6)	1827 (12.4)	
Place of recreation	1375 (3.0)	249 (3.1)	312 (3.0)	373 (3.0)	441 (3.0)	
Other	1149 (2.5)	245 (3.0)	240 (2.3)	310 (2.5)	354 (2.4)	
Initiated CPR						
Layperson	17 150 (37.3)	2941 (36.2)	3956 (37.6)	4687 (37.2)	5566 (37.8)	<.001
First responder	12 107 (26.4)	1986 (24.5)	2611 (24.8)	3463 (27.5)	4047 (27.5)	
Responding EMS	16 567 (36.1)	3165 (39.0)	3909 (37.2)	4418 (35.1)	5075 (34.5)	
First documented rhythm						
VT or VF	16 615 (36.2)	3010 (37.1)	3814 (36.3)	4523 (35.9)	5268 (35.8)	.06
PEA or asystole	29 320 (63.8)	5109 (62.9)	6698 (63.7)	8068 (64.1)	9445 (64.2)	
Advanced airway						
Oral or nasal ET	18 357 (40.0)	3456 (42.6)	4284 (40.8)	5114 (40.6)	5503 (37.4)	<.001
Supraglottic airway device	9882 (21.5)	1586 (19.5)	2334 (22.2)	2666 (21.2)	3296 (22.4)	
Other	705 (1.5)	52 (0.6)	72 (0.7)	139 (1.1)	442 (3.0)	
None	9619 (20.9)	1471 (18.1)	2066 (19.7)	2618 (20.8)	3464 (23.5)	
Unknown	7372 (16.0)	1554 (19.1)	1756 (16.7)	2054 (16.3)	2008 (13.6)	

Abbreviations: CPR, cardiopulmonary resuscitation; EMS, emergency medical services; ET, endotracheal tube; PEA, pulseless electrical activity; VF, ventricular fibrillation; VT, ventricular tachycardia.

In our exploratory analysis, we first describe risk-adjusted trends in patient survival to hospital discharge using a generalized linear mixed model with calendar year as a categorical variable and the same covariates used in our models for risk-adjusted use of hypothermia. To estimate the mean causal mediation effects of hypothermia on trends in patient survival to discharge, because both the outcome and mediator were binary variables, we applied the causal mediation effects method^10^ in R package Mediation,^11^ while accounting for clustering at the hospital level (R Foundation for Statistical Computing).

In additional exploratory analyses, we evaluated neurologic outcomes as assessed by cerebral performance category (CPC). A CPC score of 1 reflects mild or no neurologic disability, 2 reflects moderate neurologic disability, 3 reflects severe neurologic disability, 4 indicates coma or vegetative state, and 5 indicates brain death.^12^ We evaluated CPC score as a binary outcome (1 vs >1). We first fit a generalized linear mixed model on CPC score, with time as categorical predictor, adjusted for the same set of covariates as our prior models. The result showed that the probability of good cerebral performance was not varied over time (*P* = .79 for type III test on time). Mediation analysis on the trend in CPC score would not be appropriate in this setting and thus was not conducted.

## Results

Our final analytic cohort consisted of 45 935 patients with out-of-hospital cardiac arrest who survived to admission at 649 hospitals. Consistent with an increasing number of sites participating in CARES over time, the number of out-of-hospital cardiac arrests with survival to hospital admission was 8119 in 2013, 10 512 in 2014, 12 591 in 2015, and 14 713 in 2016. In total, there were 17 515 women and 28 420 men and the mean (SD) age was 59.3 (18.3) years. Minimal changes were noted over time in patient demographics and cardiac arrest characteristics (Table 1).

**Figure 2.**
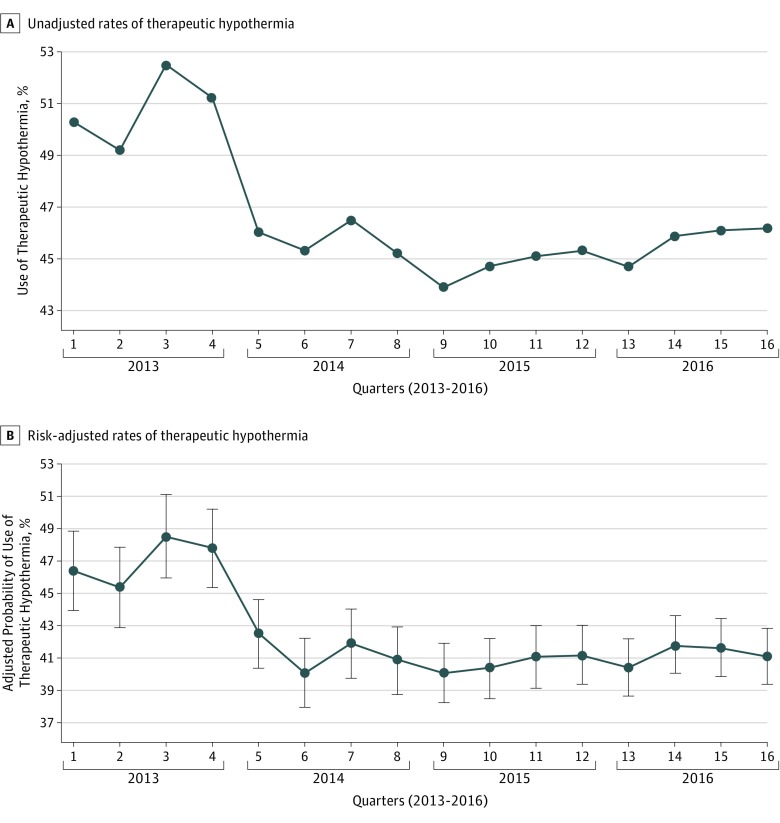
Therapeutic Hypothermia Use Over Time A, Unadjusted rates of the use of therapeutic hypothermia in the overall cohort. B, Risk-adjusted rates of the use of therapeutic hypothermia in the overall cohort. Error bars represent the SE of the point estimates.

During the study period, the overall proportion of patients receiving therapeutic hypothermia after hospital admission was 46.4%. In the first quarter of 2013, a total of 50.3% of all patients in the cohort received therapeutic hypothermia on hospital admission (Figure 1). The use of therapeutic hypothermia dropped from 52.5% in the last quarter of 2013 to 46.0% in the first quarter of 2014, after the December 2013 publication of the Targeted Temperture Management trial, and remained below 46.5% through 2016 (Figure 2A). Risk-adjusted analyses demonstrated similar findings (Figure 2B), with the risk-adjusted odds of the use of therapeutic hypothermia being significantly lower in the first quarter of 2014 through 2015 as compared with the first quarter of 2013 (Table 2). In interrupted time series, there was no significant linear trend in the risk-adjusted use of therapeutic hypothermia from the first quarter of 2013 to the last quarter of 2013, as well as from 2014 to 2015. However, the odds of therapeutic hypothermia use decreased by 18% (OR 0.82; 95% CI, 0.71-0.94; *P* = .006) between the end of 2013 and the beginning of 2014 (Table 2).

**Table 2.** Therapeutic Hypothermia Use Over Time by Interrupted Time Series Analysis

Time Period	Adjusted OR (95% CI)	*P* Value
Overall cohort		
Quarterly change in 2013	1.02 (0.98-1.07)	.32
Change between last quarter of 2013 and first quarter of 2014	0.82 (0.71-0.94)	.006
Quarterly change in 2014-2016	1.00 (0.94-1.06)	.96
VT or VF presenting rhythm		
Quarterly change in 2013	1.04 (0.97-1.11)	.28
Change between last quarter of 2013 and first quarter of 2014	0.89 (0.71-1.13)	.35
Quarterly change in 2014-2016	1.00 (0.90-1.10)	.93
PEA or asystole presenting rhythm		
Quarterly change in 2013	0.99 (0.94-1.04)	.71
Change between last quarter of 2013 and first quarter of 2014	0.75 (0.63-0.89)	.001
Quarterly change in 2014-2016	1.00 (0.85-1.17)	.99

Abbreviations: OR, odds ratio; PEA, pulseless electrical activity; VF, ventricular fibrillation; VT, ventricular tachycardia.

**Figure 3.**
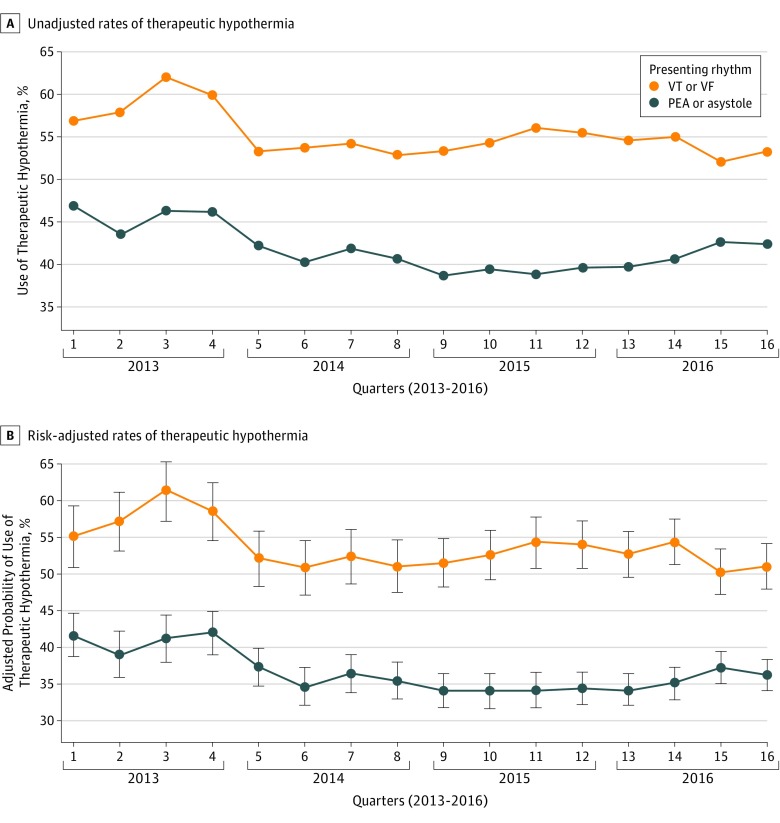
Therapeutic Hypothermia Use Over Time by Presenting Rhythm A, Unadjusted rates of use. B, Risk-adjusted rates of use. PEA indicates pulseless electrical activity; VF, ventricular fibrillation; and VT, ventricular tachycardia. Error bars represent the SE of the point estimates.

In unadjusted analyses stratified on presenting rhythm, the overall use of therapeutic hypothermia was 55.0% among patients with VT or VF and 41.5% among patients with PEA or asystole. In patients with VT or VF, the use of therapeutic hypothermia dropped from 59.9% in the last quarter of 2013 to 53.3% in the first quarter of 2014 and remained below 56.0% through 2016 (Figure 3A). Among patients with PEA or asystole, the use of therapeutic hypothermia decreased from 46.1% in the last quarter of 2013 to 42.2% in the first quarter of 2014 and remained below 42.6% through 2016. Risk-adjusted analyses demonstrated similar findings (Figure 3B). Similar point-estimate changes over time were observed for VT or VF (OR, 0.89; 95% CI, 0.71-1.13; *P* = .35) and PEA or asystole (OR, 0.75; 95% CI, 0.63-0.89; *P* = .001) from interrupted time series analysis (Table 2), and we found no evidence of interaction on presenting rhythm. Our sensitivity analysis of the 300 hospitals continuously participating in CARES throughout the study period demonstrated similar findings (eTables 1 and 2 in the Supplement).

Among patients surviving to hospital admission, overall patient survival to discharge was 38.4% and varied minimally by year (38.9% in 2013, 39.6% in 2014, 37.5% in 2015, and 38.0% in 2016). Overall, there was a small but statistically significant decreasing trend in risk-adjusted patient survival during the study period: 36.9% in 2013, 37.5% in 2014, 34.8% in 2015, and 34.3% in 2016 (*P* < .001). Mediation analysis at the patient level found that decreasing use of therapeutic hypothermia was associated with a statistically significant 0.3% increase in patient survival (eTable 3 in the Supplement), while mediation analysis at the hospital level found that decreasing use of therapeutic hypothermia was not associated with patient survival (eTable 4 in the Supplement). As such, the decreasing trend in risk-adjusted patient survival to discharge was not consistently explained by decreasing use of therapeutic hypothermia.

## Discussion

In a large, prospective registry of out-of-hospital cardiac arrest in US communities, we found that the use of therapeutic hypothermia on hospital admission decreased from 52.5% in the last quarter of 2013 to 46.0% in the first quarter of 2014 after the December 2013 publication of the TTM trial that supported more lenient target temperatures in therapeutic hypothermia. Temporal declines in the use of therapeutic hypothermia were observed for both patients with VT or VF arrest and those with PEA or asystole, suggesting that these declines were reflective of a change in practice rather than a change in patient population. During this period, overall risk-adjusted patient survival to discharge among patients admitted to the hospital decreased slightly, although decreasing trends in survival were not consistently explained by decreasing use of therapeutic hypothermia.

Therapeutic hypothermia after cardiac arrest is thought to improve patient outcomes through several mechanisms,^2,13,14^ including avoidance of fever.^3,15,16^ Accordingly, the TTM trial sought to refine our understanding of the temperature targets associated with optimal outcomes of cardiac arrest.^5^ Although this trial supports a more lenient temperature threshold, the temperature evaluated in this trial continued to require the use of cooling techniques. Management approaches that are limited to avoidance of fever alone (ie, scheduled administration of acetaminophen) have not been studied in comparison with these cooling strategies.

Prior studies have shown low rates of the use of therapeutic hypothermia.^17,18^ A lack of familiarity with and availability of protocols for therapeutic hypothermia have been reported among the most frequent barriers to the use of therapeutic hypothermia.^19,20^ Additional barriers include availability of equipment and high workload demands for nursing staff.^19,20^ Strategies that support a more lenient temperature threshold may address some of these barriers. However, the results of our study suggest that the targeted TTM trial may have been inaccurately interpreted in clinical practice as demonstrating that therapeutic hypothermia lacks benefit in the management of cardiac arrest.

The CARES registry defines therapeutic hypothermia as the provision of measures to reduce the patient’s body temperature by either noninvasive or invasive means, regardless of temperature goal. Accordingly, the observed temporal decline in the use of therapeutic hypothermia reflects a real practice change, rather than a change in terminology from *therapeutic hypothermia* to *targeted temperature management*. Although a temporal decrease in patient-level and hospital-level survival was not explained by a decrease in the use of therapeutic hypothermia, these analyses were underpowered owing to a small number of participating hospitals. Furthermore, we are unable to comment on potential changes in the quality of therapeutic hypothermia (eg, time to target temperature and time in target temperature) that may also affect patient survival. The decrease in survival that was noted in this study is in contrast to prior studies that have reported improving trends in patient survival for out-of-hospital cardiac arrest in time periods that preceded this study^9,21,22^ and raises the possibility that a decrease in the use and quality of therapeutic hypothermia has negatively affected patient survival.

Prior research has shown that studies with negative findings can have immediate, though often small, effects on clinical practice.^23,24,25^ Although the TTM trial did not have negative findings, it appears to have been interpreted as such in clinical practice, with changes in the use of therapeutic hypothermia similar to the immediate change in use of an intra-aortic balloon pump for cardiogenic shock and thrombectomy for acute myocardial infarction after trials with negative findings.^23,25^ The effect of the TTM trial may have been furthered by near concurrent publication of a study with negative findings for prehospital therapeutic hypothermia.^26^ Although one potential explanation for our findings would be associated with a reduction in the use of therapeutic hypothermia for clinical scenarios for which supporting data are less robust (ie, PEA or asystolic arrests), we found no evidence of interaction on presenting rhythm to support this interpretation. The observed change in practice may reflect early adopters of these data and/or those who were tenuously committed to the use of therapeutic hypothermia and ready to accept data that supported a change in practice. This change is further suggested by the lack of continued decline in hypothermia use after the one-time change after publication of the TTM trial.

### Strengths and Limitations

A strength of our study is the use of data from a large, national, clinical registry of patients with out-of-hospital cardiac arrest. However, several study limitations should be considered. First, CARES was designed to support public health surveillance of cardiac arrest and minimize burdens to participation.^6^ Accordingly, we do not have access to data on all patient factors (eg, patient comorbidities and neurologic status on arrival) and resuscitation factors (eg, use of epinephrine, quality of cardiopulmonary resuscitation) that may influence the use of therapeutic hypothermia and patient survival. However, there were minimal differences in patient or cardiac arrest characteristics over time, suggesting that observed trends in the use of therapeutic hypothermia and patient survival are unlikely to be a function of changing severity of patient illness. Second, our analysis of trends in the use of therapeutic hypothermia should be strictly interpreted as reflecting the experience of hospitals participating in the CARES registry with at least 10 out-of-hospital cardiac arrests over a 4-year period. Given differences in the estimated US incidence of out-of-hospital cardiac arrest from CARES as compared with other registries,^27^ this has implications for the generalizability of the findings. Third, our study excluded patients with out-of-hospital cardiac arrest occurring in facilities with medical professionals. This exclusion was to ensure that events analyzed reflected out-of-hospital cardiac arrest, given the lack of trial evidence to support the use of therapeutic hypothermia for in-hospital cardiac arrest. Future analysis of trends in the use of therapeutic hypothermia for in-hospital cardiac arrest could address this question. Finally, we lack data on the temperature achieved among patients who were managed with therapeutic hypothermia to further inform the potential effects of the TTM trial on the approach to management of out-of-hospital cardiac arrest.

## Conclusions

The use of therapeutic hypothermia decreased in a large US registry of patients with out-of-hospital cardiac arrest soon after the publication of a study supporting more lenient temperature thresholds. Concurrent with this change, overall survival of cardiac arrest among patients admitted to the hospital decreased, but survival was not explained by patient-level or hospital-level trends in the use of therapeutic hypothermia. These findings raise concern for a potential change in clinical practice away from guideline-recommended active cooling after out-of-hospital cardiac arrest.

## Notes

Supplement. **eAppendix.** Methods **eTable 1.** Risk-Adjusted Odds of Therapeutic Hypothermia Use Over Time (First Quarter 2013 as Reference) Among Hospitals With Continuous Participation in CARES **eTable 2.** Therapeutic Hypothermia Use Over Time by Interrupted Time Series Analysis Among Hospitals With Continuous Participation in CARES **eTable 3**. Estimated Causal Effects for Patient-Level Analysis **eTable 4**. Estimated Causal Effects for Hospital-Level Analysis
