# Supplementary material for: Temporal Trends in the Use of Therapeutic Hypothermia for Out-of-Hospital Cardiac Arrest
Source: JAMA Netw Open. 2018 Nov 9;1(7):e184511. doi: 10.1001/jamanetworkopen.2018.4511 (PMC6324404; doi:10.1001/jamanetworkopen.2018.4511)
Supplement: Supplement. — eAppendix. Methods eTable 1. Risk-Adjusted Odds of Therapeutic Hypothermia Use Over Time (First Quarter 2013 as Reference) Among Hospitals With Continuous Participation in CARES eTable 2. Therapeutic Hypothermia Use Over Time by Interrupted Time Series Analysis Among Hospitals With Continuous Participation in CARES eTable 3. Estimated Causal Effects for Patient-Level Analysis eTable 4. Estimated Causal Effects for Hospital-Level Analysis [file jamanetwopen-1-e184511-s001.pdf]

Supplementary Online Content

Bradley SM, Liu W, McNally B, et al; Cardiac Arrest Registry to Enhance Survival (CARES) Surveillance Group. Temporal trends in the use of therapeutic hypothermia for out-of-hospital cardiac arrest. *JAMA Netw Open.* 2018;1(7):e184511. doi:10.1001/jamanetworkopen.2018.4511

eAppendix. Methods
eTable 1. Risk-Adjusted Odds of Therapeutic Hypothermia Use Over Time (First Quarter 2013 as Reference) Among Hospitals With Continuous Participation in CARES
eTable 2. Therapeutic Hypothermia Use Over Time by Interrupted Time Series Analysis Among Hospitals With Continuous Participation in CARES
eTable 3. Estimated Causal Effects for Patient-Level Analysis
eTable 4. Estimated Causal Effects for Hospital-Level Analysis

This supplementary material has been provided by the authors to give readers additional information about their work.

eAppendix. Methods

Analyses for temporal trends in survival outcomes mediated by trends in hypothermia use

We used causal mediation effects method,[9] fit in R package “Mediation”[10] to estimate the average causal mediation effects of hypothermia on the trend of survival to discharge. We performed these analyses separately at the patient-level and hospital-level. Multilevel models were used to take into account the heterogeneity within and between cluster levels. Specifically, we fit hospital random effect logistic model for both mediator and outcome, using centering within cluster (CWC) method[22] to decompose the effect of mediator on the outcome into between and within cluster effects. Random hospital intercept and random hospital time slope were included in both outcome and mediator models, while using time as a categorical variable. The same set of covariates was adjusted in both mediator and outcome models. Quasi-Bayesian Monte Carlo approximation was used to simulate the model parameters from their sampling distribution. The estimated average causal mediation effect represents the expected difference in the survival to hospital discharge when the mediator (therapeutic hypothermia) took the potential value when controlling for the time. The average direct effect represents the expected difference in the survival to hospital discharge when the treatment is changed but the mediator is held constant at the potential value.

© 2018 Bradley SM et al. *JAMA Network Open.*

eTable 1. Risk-Adjusted Odds of Therapeutic Hypothermia Use Over Time (First Quarter 2013 as Reference) Among Hospitals With Continuous Participation in CARES

**Overall Cohort**															
**Year**	**2013**			**2014**				**2015**				**2016**			
**Quarter**	**2**	**3**	**4**	**1**	**2**	**3**	**4**	**1**	**2**	**3**	**4**	**1**	**2**	**3**	**4**
**OR**	0.95	1.08	1.03	0.85	0.76	0.85	0.79	0.77	0.78	0.80	0.78	0.77	0.79	0.80	0.74
**95% CL**	(0.82, 1.09)	(0.93, 1.25)	(0.9, 1.19)	(0.74, 0.98)	(0.66, 0.87)	(0.74, 0.98)	(0.68, 0.9)	(0.67, 0.88)	(0.68, 0.9)	(0.69, 0.92)	(0.68, 0.89)	(0.67, 0.88)	(0.69, 0.9)	(0.7, 0.92)	(0.64, 0.84)
**P-value**	0.46	0.33	0.65	0.02	<.001	0.02	<.001	<.001	<.001	0.002	0.0004	<.001	<.001	<.001	<.001
**PEA/Asystole**															
**Year**	**2013**			**2014**				**2015**				**2016**			
**Quarter**	**2**	**3**	**4**	**1**	**2**	**3**	**4**	**1**	**2**	**3**	**4**	**1**	**2**	**3**	**4**
**OR**	0.87	0.93	0.99	0.85	0.71	0.82	0.75	0.72	0.72	0.72	0.70	0.70	0.71	0.77	0.74
**95% CL**	(0.72, 1.04)	(0.77, 1.11)	(0.83, 1.18)	(0.71, 1)	(0.6, 0.84)	(0.69, 0.98)	(0.63, 0.9)	(0.61, 0.86)	(0.61, 0.86)	(0.6, 0.85)	(0.6, 0.83)	(0.6, 0.83)	(0.6, 0.84)	(0.65, 0.91)	(0.63, 0.87)
**P value**	0.12	0.40	0.94	0.06	<.001	0.03	0.001	<.001	<.001	<.001	<.001	<.001	<.001	<.001	<.001
**VT/VF Presenting Rhythm**															
**Year**	**2013**			**2014**				**2015**				**2016**			
**Quarter**	**2**	**3**	**4**	**1**	**2**	**3**	**4**	**1**	**2**	**3**	**4**	**1**	**2**	**3**	**4**
**OR**	1.12	1.42	1.13	0.86	0.88	0.91	0.86	0.88	0.91	0.99	0.96	0.91	0.97	0.88	0.75
**95% CL**	(0.88, 1.43)	(1.11, 1.82)	(0.89, 1.44)	(0.68, 1.1)	(0.69, 1.11)	(0.72, 1.16)	(0.68, 1.09)	(0.7, 1.11)	(0.72, 1.15)	(0.78, 1.25)	(0.76, 1.21)	(0.72, 1.15)	(0.77, 1.22)	(0.7, 1.11)	(0.59, 0.94)
**P value**	0.35	0.006	0.32	0.23	0.27	0.46	0.22	0.29	0.43	0.91	0.72	0.44	0.77	0.29	0.01

© 2018 Bradley SM et al. *JAMA Network Open.*

eTable 2. Therapeutic Hypothermia Use Over Time by Interrupted Time Series Analysis Among Hospitals With Continuous Participation in CARES

**Overall Cohort**	**Adjusted OR**	**95% CI**		**P value**
Quarterly change in 2013	1.02	(0.98,	1.07)	0.33
Change between last quarter 2013 and first quarter of 2014	0.88	(0.75,	1.02)	0.09
Quarterly change in 2014-2016	0.99	(0.93,	1.06)	0.85
**VT/VF Presenting Rhythm**	**Adjusted OR**	**95% CI**		**P value**
Quarterly change in 2013	1.06	(0.98 ,	1.14)	0.15
Change between last quarter 2013 and first quarter of 2014	0.91	(0.70 ,	1.17)	0.46
Quarterly change in 2014-2016	1.00	(0.89 ,	1.11)	0.97
**PEA/Asystole Presenting Rhythm**	**Adjusted OR**	**95% CI**		**P-value**
Quarterly change in 2013	1.00	(0.94 ,	1.06)	0.96
Change between last quarter 2013 and first quarter of 2014	0.85	(0.71 ,	1.03)	0.10
Quarterly change in 2014-2016	0.99	(0.83 ,	1.18)	0.93

© 2018 Bradley SM et al. *JAMA Network Open.*

eTable 3. Estimated Causal Effects for Patient-Level Analysis

	Total effect				ACME				ADE (Average direct effect)			
**Year**	**estimate**	**95% CI**		**p-value**	**estimate**	**95% CI**		**p-value**	**estimate**	**95% CI**		**p-value**
2014	0.007	-0.006,	0.021	0.322	0.003	0.002,	0.004	<.0001	0.004	-0.009,	0.019	0.586
2015	-0.015	-0.028,	-0.002	0.026	0.003	0.002,	0.004	<.0001	-0.019	-0.031,	-0.005	0.006
2016	-0.019	-0.033,	-0.006	0.008	0.003	0.002,	0.004	<.0001	-0.023	-0.037,	-0.009	0.002

eTable 3 shows the results of the causal effects estimates for the patient-level analysis. Using year 2013 as a reference, in 2014 the probability of survival to discharge increased 0.7% overall with 0.4% attributable to temporal trends (average direct effect) and 0.3% attributable to decreasing hypothermia use over time (average causal mediation effect). The total effect and direct effect of time on survival to discharge were not significant from 2013 to 2014, but the indirect effect of time to increase the survival probability through decreasing hypothermia over time was significant (p<0.0001). The average causal mediation effect estimates were similar for year 2015 and 2016. However the total effect and direct effect of time on survival were in different directions in 2015 and 2016. Comparing to 2013, the overall survival probability decreased 1.5% (95%CI; -0.2%, -2.8%, p=0.03) at 2015, and decreased 1.9% (95%CI; -0.6%, -3.3%, p=0.008) at 2016. The direct effect temporal trends on survival were also negative, decreased 1.9% at 2015, and deceased 2.3% at 2016, both were significant.

© 2018 Bradley SM et al. *JAMA Network Open.*

eTable 4. Estimated Causal Effects for Hospital-Level Analysis

	Total effect				ACME				ADE (Average direct effect)			
**Year**	**estimate**	**95% CI**		**p-value**	**estimate**	**95% CI**		**p-value**	**estimate**	**95% CI**		**p-value**
2014	0.007	-0.007 ,	0.02	0.35	-0.002	-0.006 ,	0.002	0.45	0.008	-0.005 ,	0.021	0.21
2015	-0.016	-0.029 ,	-0.003	0.01	-0.002	-0.006 ,	0.003	0.44	-0.014	-0.028 ,	-0.001	0.04
2016	-0.019	-0.032 ,	-0.006	0.001	-0.001	-0.005 ,	0.002	0.40	-0.018	-0.03 ,	-0.004	0.01

eTable 4 shows the results of the causal effects estimates for the hospital-level analysis. Using year 2013 as a reference, in 2014 the probability of survival to discharge increased 0.7% overall with all of this increases attributable to temporal trends (average direct effect) and a 0.2% decrease in survival attributable to decreasing hypothermia use over time, though these effects were not statistically significant. The ACME estimates continued to demonstrate an attribution of decreasing survival to decreasing use of hypothermia in 2015 and 2016, thought these estimated causal effects were not statistically significant.

© 2018 Bradley SM et al. *JAMA Network Open.*
